# Complete Genomic Sequence of “*Thermofilum adornatus*” Strain 1910b^T^, a Hyperthermophilic Anaerobic Organotrophic Crenarchaeon

**DOI:** 10.1128/genomeA.00726-13

**Published:** 2013-09-12

**Authors:** I. N. Dominova, I. V. Kublanov, O. A. Podosokorskaya, K. S. Derbikova, M. V. Patrushev, S. V. Toshchakov

**Affiliations:** Immanuel Kant Baltic Federal University, Kaliningrad, Russiaa; Winogradsky Institute of Microbiology, Russian Academy of Sciences, Moscow, Russiab

## Abstract

The complete genomic sequence of a novel hyperthermophilic crenarchaeon, strain 1910b^T^, was determined. The genome comprises a 1,750,259-bp circular chromosome containing single copies of 3 rRNA genes, 43 tRNA genes, and 1,896 protein-coding sequences. *In silico* genome-genome hybridization suggests the proposal of a novel species, “*Thermofilum adornatus*” strain 1910b^T^.

## GENOME ANNOUNCEMENT

*Thermofilaceae* (1) is one of two families of the crenarchaeal order *Thermoproteales*, represented by a sole valid species, *Thermofilum pendens*, as well as several nonvalidated strains and uncultured clones. This family represents a deep phylogenetic lineage within the order *Thermoproteales*, possibly being an individual order in a crenarchaeal class *Thermoprotei*. Isolated from a solfatara in Iceland (2), *T. pendens* is a hyperthermophilic, moderately acidophilic, sulfur-dependent anaerobic heterotroph, which has an obligatory need for the *Thermoproteus tenax* polar lipid fraction for growth. Results of its genome analysis (3) revealed a number of proteins involved in peptide and saccharide utilization, while biosynthetic pathways for purines, most amino acids, and cofactors were absent, indicating the adaptation to life in organic-rich environments and dependence on other organisms providing lacking nutrients. Notably, the nonvalidated species “*Thermoproteus librum*,” which has a 16S rRNA gene sequence that is 100% identical, does not require the addition of cell components of other organisms (4).

Strain 1910b^T^ was isolated from a mud sample from a black mud pit (86°C, pH 5.5) located near Pauzhetka (Kamchatka Peninsula, Russia). The strain grows optimally at 92°C and pH 6.0 to 6.5 in the presence of *Fervidicoccus fontis* strain 1910a culture broth with glucose and peptone as the substrates.

A BLAST search (5) revealed 97.3% 16S rRNA gene sequence similarity between strain 1910b^T^ and *T. pendens* strain Hvv3^T^.

For the genome sequencing of strain 1910b^T^, we used a combination of fragment and mate-paired library approaches. The fragment library was sequenced with the Illumina MiSeq system, and the mate-paired library, with an average insert length of 2,200 bp, was sequenced with the Life Technologies PGM system. A total of 0.5 million Illumina paired-end reads were trimmed and corrected with the Quake sequencing error correction tool (6), and 3.1 million 200-bp PGM mate-paired reads were split and trimmed by use of the CLC Genomics Workbench and then were subjected to error correction with the SAET tool (7).

Reads were assembled with CLC Assembler using recommended parameters (8). Obtained contigs were scaffolded with SSPACE (9) and remaining gaps were closed with the GapFiller tool (10), resulting in one 1.75-Mb contig. For circularization, we split the contig into 2 parts and joined the resulting sequences in reverse order with a 500-bp gap. This “artificial” gap was successfully closed with GapFiller, proving that the obtained contig corresponds to the circular chromosome of strain 1910b^T^.

The genome of strain 1910b^T^ is comprised of a 1,750,259-bp circular chromosome with a G+C content of 46.5%. Annotation of the genome was performed with the NCBI Prokaryotic Genomes Annotation Pipeline with subsequent manual curation.

The chromosome of strain 1910b^T^ contains single rRNA copies, of which the 16S and 23S rRNAs were found together, while the 5S rRNA was found to be located in another region, as was also shown for the *T. pendens* strain Hrk5 genome (3). Forty-three tRNA genes and 1,896 protein-coding sequences were found. *In silico* genome-genome hybridization performed using the GGDC 2.0 algorithm (11) revealed a 0% probability that strain 1910b^T^ and *T. pendens* strain Hrk5 (= strain Hvv3^T^) represent the same species. Based on this evidence, we assume that strain 1910b^T^ represents a novel species “*Thermofilum adornatus*.” Its complete description will be published elsewhere.

### Nucleotide sequence accession number.

The genome sequence of “*Thermofilum adornatus*” strain 1910b^T^ has been deposited in NCBI GenBank under the accession number CP006646.
